# The determinants of mental health inequalities between Chinese migrants and non-migrants during the Shanghai 2022 lockdown: a Blinder-Oaxaca decomposition

**DOI:** 10.1186/s12939-024-02223-2

**Published:** 2024-07-09

**Authors:** Meng Zheng, Di Kong, Kunpeng Wu, Gen Li, Yi Zhang, Wen Chen, Brian J. Hall

**Affiliations:** 1 Department of Medical Statistics, School of Public Health, Sun Yat-sen University, Guangzhou, China; 2 Center for Migrant Health Policy, Sun Yat-sen University, Guangzhou, China; 3 Center for Global Healthy Equity, NYU Shanghai, Shanghai, China

**Keywords:** Mental Health, Health inequalities, Social determinants of Health, Emergency Psychiatry, Lockdown, Blinder-Oaxaca decomposition

## Abstract

**Background:**

The mental health inequality between migrants and non-migrants was exacerbated by the COVID-19 pandemic. Identifying key determinants of this inequality is essential in promoting health equity.

**Methods:**

This cross-sectional study recruited Shanghai residents by purposive sampling during the city-wide lockdown (from April 29 to June 1, 2022) using an online questionnaire. Migration statuses (non-migrants, permanent migrants, and temporary migrants) were identified by migration experience and by household registration in Shanghai. Mental health symptoms (depression, anxiety, loneliness, and problematic anger) were assessed by self-report scales. The nonlinear Blinder-Oaxaca decomposition was used to quantify mental health inequality (i.e., differences in predicted probabilities between migration groups) and the contribution of expected correlates (i.e., change in predicted probability associated with variation in the correlate divided by the group difference).

**Results:**

The study included 2738 participants (771 [28.2%] non-migrants; 389 [14.2%] permanent migrants; 1578 [57.6%] temporary migrants). We found inequalities in depression (7.1%) and problematic anger (7.8%) between permanent migrants and non-migrants, and inequalities in anxiety (7.3%) and loneliness (11.3%) between temporary migrants and non-migrants. When comparing permanent migrants and non-migrants, age and social capital explained 12.7% and 17.1% of the inequality in depression, and 13.3% and 21.4% of the inequality in problematic anger. Between temporary migrants and non-migrants, age and social capital also significantly contributed to anxiety inequality (23.0% and 18.2%) and loneliness inequality (26.5% and 16.3%), while monthly household income (20.4%) and loss of monthly household income (34.0%) contributed the most to anxiety inequality.

**Conclusions:**

Significant inequalities in depression and problematic anger among permanent migrants and inequalities in anxiety and loneliness among temporary migrants were observed. Strengthening social capital and economic security can aid in public health emergency preparedness and promote mental health equity among migrant populations.

**Supplementary Information:**

The online version contains supplementary material available at 10.1186/s12939-024-02223-2.

## Background

The COVID-19 pandemic created an increased global burden of mental disorders. An estimated 53.2 million cases of major depression (an increase of 27.6%) and 76.2 million cases of anxiety (an increase of 25.6%) resulted from the COVID-19 pandemic globally [1]. Addressing mental health inequality is a key goal for global mental health and sustainable development, especially during the pandemic [2]. Following the definition of health inequality, mental health inequality is the avoidable difference in mental health status and determinants between disadvantaged and more advantaged social groups [3].

It is well-established that migration is related to disadvantaged circumstances and mental health outcomes. Compared to non-migrants, migrants encounter greater exposure to stressors and to the social determinants known to exacerbate mental health problems, and therefore, may experience worse mental health [4]. Chinese citizens who are living in another city other than the city where they have household registration (*hukou* in Chinese, managed at the municipal level) are classified as internal migrants [5]. Since the *hukou* affiliation is linked with the provision of public social services (e.g., housing, education, job benefits, health insurance, employment) [6], and marginalization, isolation, and discrimination [7], internal migrants without a *hukou* (temporary migrants) are more vulnerable to mental health problems than migrants with a *hukou* (permanent migrants) [8]. Individuals with higher socioeconomic status (SES), including higher education and income, are likely to acquire a *hukou* in their migration destination, but they may face additional stressors such as purchasing property [9]. Thus, we hypothesized that differences in key social determinants, including SES, may lead to heterogeneity among internal migrants in mental health.

The current literature is limited since the majority of evidence on mental health inequalities between internal migrants and non-migrants relies on studies with simple classification based on their current place of usual residence, regardless of their migration experience [10, 11]. Our previous work among middle-aged and elderly migrants in China demonstrated that different types of internal migrants have heterogenous mental health outcomes. We found a significantly lower incidence of depression in permanent migrants than non-migrants [12]. However, there remains limited evidence from younger migrants, which includes a larger proportion of internal migrants than the middle-aged and elderly [13], and it is unclear whether mental health inequality among people with different migration statuses was exacerbated by the scarcity of public service resources during pandemic-era lockdowns.

In addition, although previous studies document the association between social determinants and mental health (e.g., education, employment, social capital, physical health, and household factors) [14, 15], few studies have quantified and compared the contributions of these factors to mental health inequality. Identifying the determinants of differences in health status is critical for population-based mitigation strategies, and in turn contribute to achieving the 2030 Agenda for Sustainable Development Goal to leave no one behind [16].

Shanghai is the largest city in China and has one of the largest internal migrant populations − 10.5 million - which accounts for 42.1% of its total population [17]. We conducted an online cross-sectional study among Shanghai residents with different migration statuses (non-migrants, permanent migrants, and temporary migrants) during the sudden city-wide COVID-19 lockdown in 2022. This study aimed to (1) compare the mental health conditions among Chinese adult migrants and non-migrants during the lockdown, and assess possible mental health inequalities between groups, and (2) identify the major social determinants of mental health inequality observed between migrants and non-migrants.

## Methods

### Study design and participants

A cross-sectional survey was conducted in Shanghai during the COVID-19 lockdown in 2022 (from April 29 to June 1). An unexpected, unprecedented and complete citywide lockdown was implemented among the entire population of 24.9 million from March 28, 2022 to June 1, 2022. In this survey, Chinese adult citizens (≥ 18 years old) living in 16 administrative districts of Shanghai were recruited by purposive sampling. International migrants were not included in this study, as the *hukou* system only applies to Chinese citizens. The survey company Wenjuanxing randomly recruited individuals via WeChat with the aim for 200 persons per district. Sampling was concluded when the quota or the end of the lockdown was reached. Participants completing the survey received a 6 Chinese Yuan incentive (approximately 1 USD). Network IP address was checked and three filter questions with predetermined options were included to enhance the data quality of the online questionnaire. The questionnaire was brief and completed in less than 15 min. A total of 3,230 individuals provided valid responses. Ten participants with data missing on sex and 407 students (who are not included in China Migrants Dynamic Survey definition of migrants [18] and had stable supplies from universities or at home during the lockdown) were excluded from our study.

Additionally, we excluded 75 elderly participants (aged 60 and above) from the analyses since we were comparing migrant to non-migrants who were working age and living in Shanghai. Elders may not represent a valid group for these study comparisons. The final sample consist of 2,738 participants. All respondents completed the consent form and explicitly agreed to participate. The study was approved by the Institutional Review Board of the NYU Shanghai (2022-008-NYUSH).

### Measurement

#### Mental health outcomes

Mental health outcomes included depression, anxiety, loneliness, and problematic anger, which are the most common mental health problems reported during the pandemic [19].

Depression symptoms in the past two weeks were measured by the Patient Health Questionnaire (PHQ-9), one of the most widely used screening instruments. It has nine items with each scoring 0–3 and a total score ranging from 0 to 27, where score of 10 or above indicates possible major depression [20]. Thus, the indicator of depression was coded as “0” = no (scoring 0–9), “1” = yes (scoring 10 or above). The Cronbach’s alpha in the current study was 0.89.

Anxiety symptoms in the past two weeks were measured by the seven-item Generalized Anxiety Disorder Scale (GAD-7). Participants answered on a four-point Likert scale (0 = not at all, 3 = nearly every day). A summed score of 0–21 was generated to illustrate the severity of symptoms, with a higher score indicating worse severity and a cut-off of 10 for moderate to severe anxiety [21]. Therefore, anxiety was coded as “0” = no (scoring 0–9), “1” = yes (scoring 10 or above). The Cronbach’s alpha in the study was 0.92.

Loneliness was assessed by the 3-item version of UCLA Loneliness Scale. The UCLA Loneliness Scale was used widely in Chinese migrants. Participants were asked about the frequency they feel lack of companionship, left out, and isolated from others. Each item was rated on 3-point scale (1 = hardly ever or never, 3 = almost always), giving a total score of 3–9, which was dichotomized into “0” = not lonely (scoring 3 to 5) and “1” = lonely (scoring 6 or above) [22]. The Cronbach’s alpha in the current study was 0.88.

The Dimensions of Anger Reactions (DAR-5) was applied to assess anger experiences in the past four weeks. It is composed of four anger response parameter items (including frequency, intensity, duration, and antagonism) as well as one item for social relationship impairment. Each item was answered on a 5-point Likert scale, giving a score range from 5 to 25. A score of 12 or above was coded as “1” = problematic anger, or “0”= no otherwise [23]. The Cronbach’s alpha in the current study was 0.79.

#### Migration status

In this study, migration status was ascertained by both migration experience and *hukou* status to capture the heterogeneity within the migrant population by asking: “Were you born in Shanghai” and for participants not born in Shanghai, “Do you have a Shanghai *hukou*”? The former condition was used to separate non-migrants and migrants, where non-migrants were those born in Shanghai with a Shanghai *hukou*. The latter condition was employed to further divide migrants into “permanent migrants” and “temporary migrants”, defining permanent migrants as those who changed their *hukou* to Shanghai, and temporary migrants as those who live in Shanghai without a Shanghai *hukou*.

#### Social determinants

Based on previous studies [5, 15], we collected the following information as social determinants of mental health, including:

1. Individual demographics: age, sex, education level [secondary or lower, high school, college or higher], marital status [single, married/cohabitating, widowed/divorced], and employment status [yes/no]).
2. Household factors: living arrangement [living alone or not], monthly household income [≤ 4,000 Chinese Yuan, 4,001–8,000, 8,001–15,000, 15,001–30,000, ≥ 30,001], loss of monthly household income during the lockdown [none, < 50%, ≥ 50%], and partner violence) [yes/no]. Intimate partner violence was screened by the Hurt, Insult, Threaten, And Scream Scale (HITS) in married/partnered participants. The HITS screening for four types of common domestic violence on a 5-point Likert scale, and a cut-off point of 10 was used to identified possible violence (0 = no, 1 = yes) [24]. The Cronbach’s alpha in the current study was 0.95.
3. Health-related factors: COVID-19 infection in lockdown [yes/no], chronic disease [yes/no], self-rated health [relatively bad or not], past diagnosis of mental illness [yes/no], hazardous drinking[yes/no], and smoking during the lockdown [yes/no]. Hazardous drinking was evaluated with two items from the alcohol use disorders identification test. The frequency and quantity of drinking was assessed and each item was scored 0–4. A score above 0 on either item indicates a hazardous drinking [25].
4. Social capital was the sum of trustworthy network connections that empower individuals and consisting of structural, cognitive, bonding, bridging, and linking components. Bonding capital reflects individuals’ capacity to bond with others within their own community. Bridging capital indicates individual’s ability to connect with people from different communities or social identities. In this study, social capital was assessed by subscales (bonding social capital and bridging social capital) from the Chinese version of Revised Personal Social Capital Scale (PSCS-R). Responses to each question were scored on a 5-point Likert scale (1 = none, 5 = all). The Cronbach’s alpha in the current study was 0.80. Items were summed to create a total score ranging from 8 to 40, with higher scores indicating better social capital. This scale was divided into quartiles in this study. The subscales of PSCS-R are available in appendix (Table S1).

### Statistical analysis

The participants’ characteristics were described by mean ± standard deviation (SD) and frequency (proportion) of mental health outcomes and social factors. To examine the unadjusted differences in mental health outcomes among the participants with different migration statuses, we used Chi-square tests for categorical variables and Dunnett-*t* tests for continuous variables.

Bivariate and multivariable logistic regressions were employed to assess the unadjusted and adjusted associations (adjusting for individual demographics, household factors, health-related factors, and social capital) between migration statuses and mental health, with non-migrants as the reference group.

When significant migration-status-based group difference in mental health outcome was found in in the adjusted analysis, determinants of inequality were further identified by Blinder-Oaxaca (BO) decomposition [26]. The BO decomposition is a statistical tool that quantifies the extent to which an individual determinant explains a health inequality. The method has been widely used in epidemiology and health-related studies, but with few applications to mental health [27–29].

We used the nonlinear BO method in this study as the mental health outcomes were binary variables [30]. The BO decomposition analyses included two phases. Firstly, we calculated the predicted probability of mental health problems by estimating a logistic regression model for each group and then the difference in predicted probabilities between the migrant groups (i.e., permanent, or temporary migrants) and non-migrants was used to quantify the mental health inequality.

Secondly, we quantified the contribution of each social determinant, including individual demographics, household factors, health-related factors, and social capital to the inequality. In this step, we calculated the change in the predicted probability (absolute contribution) from replacing one social determinant of the reference group (non-migrants) with that of a migrant group (permanent or temporary migrants), while fixing the distribution of other determinants in the model. The proportion of explanation (relative contribution) was calculated by dividing the change in predicted probability by the group-difference in predicted probabilities. The sign of the proportion of explanation indicates the direction of the contribution, where a negative sign indicates a negative contribution, meaning that removing the intergroup differences in this factor will increase the inequality, and vice versa. BO decomposition’s statistical method was elaborated in the appendix (p3).

R 4.1.1 and STATA 11 (Stata Corp, College Station, TX) were used to perform statistical analysis. All analyses used a significance level of 0.05.

#### Role of the funding source

The funding source played no role in the study’s design, data collection, data analysis, data interpretation, report writing, or the decision to submit the manuscript for publication.

## Results

Descriptive statistics across three migration-status-based groups are presented in Table 1. A total of 2,738 Shanghai residents were included in the data analysis, including 771 (28.2%) non-migrants, 389 (14.2%) permanent migrants, and 1578 (57.6%) temporary migrants. Both permanent and temporary migrant groups were younger and presented a lower rate of chronic diseases yet had a higher rate of hazardous drinking than non-migrants (permanent:36.0% and temporary: 29.7% vs. non-migrants: 25.3%). Compared to non-migrants, permanent migrants on average had higher rates of completing college or higher level of education (permanent: 88.2% vs. non-migrants: 82.1%), employment (92.5% vs. 91.1%), more monthly household income (≥ 30,001 Chinese Yuan: 20.3% vs. 12.6%), and greater social capital (P_75_-P_100_: 30.3% vs. 23.5%). On the contrary, temporary migrants were characterized by lower education levels than non-migrants (college or higher level, temporary: 47.9% vs. non-migrants: 82.1%), lower employment (80.9% vs. 91.1%), much lower household income (≤ 4000 Chinese Yuan: 10.8% vs. 6.2%), and less social capital (P_75_-P_100_: 14.5% vs. 23.5%). Notably, temporary migrants endured significantly greater loss of income (> 50%: 51.9% vs. 14.9%) and a higher prevalence of COVID-19 infection (5.5% vs. 3.5%) than non-migrants during the lockdown.

**Table 1:** Characteristics of participants with different migration statuses

	Total sample		Non-migrants		Permanent migrants			Temporary migrants	
	*n* = 2,738		*n* = 771		*n* = 389	*p*		*n* = 1,578	*p*
Depression (yes)	748(27.3%)		159(20.6%)		108(27.8%)	0.0080		481(30.5%)	< 0.0001
Anxiety (yes)	576(21.0%)		125(16.2%)		80(20.6%)	0.080		371(23.5%)	< 0.0001
Problematic anger (yes)	1,024(37.4%)		253(32.8%)		158(40.6%)	0.011		613(38.8%)	0.005
Loneliness (yes)	903(33.0%)		201(26.1%)		112(28.8%)	0.360		590(37.4%)	< 0.0001
Age (years, Mean ± SD)	34.1 ± 9.0		37.7 ± 10.1		36.0 ± 9.4	0.0039		31.8 ± 7.6	< 0.0001
Sex (female)	1,286(47.0%)		374(48.5%)		208(53.5%)	0.125		704(44.6%)	0.083
Education level						0.0040			< 0.0001
secondary or lower	393(14.4%)		27(3.5%)		16(4.1%)			350(22.2%)	
high school	613(22.4%)		111(14.4%)		30(7.7%)			472(29.9%)	
college or higher	1,732(63.3%)		633(82.1%)		343(88.2%)			756(47.9%)	
Marital status						0.062			< 0.0001
single	741(27.1%)		178(23.1%)		85(21.9%)			478(30.3%)	
married/cohabitating	1,894(69.2%)		551(71.5%)		294(75.6%)			1049(66.5%)	
widowed/divorced	103(3.8%)		42(5.4%)		10(2.6%)			51(3.2%)	
Employment status (no)	399(14.6%)		69(8.9%)		29(7.5%)	0.452		301(19.1%)	< 0.0001
Living arrangement (living alone)	296(10.8%)		47(6.1%)		34(8.7%)	0.122		215(13.6%)	< 0.0001
Monthly household income (Chinese Yuan)						< 0.0001			< 0.0001
≤ 4,000	240(8.8%)		48(6.2%)		21(5.4%)			171(10.8%)	
4,001–8,000	756(27.6%)		150(19.5%)		47(12.1%)			559(35.4%)	
8,001–15,000	853(31.2%)		224(29.1%)		100(25.7%)			529(33.5%)	
15,001–30,000	631(23.0%)		252(32.7%)		142(36.5%)			237(15.0%)	
≥ 30,001	258(9.4%)		97(12.6%)		79(20.3%)			82(5.2%)	
Loss of monthly household income						0.845			< 0.0001
none	539(19.7%)		244(31.6%)		124(31.9%)			171(10.8%)	
< 50%	1,204(44.0%)		412(53.4%)		204(52.4%)			588(37.3%)	
≥ 50%	995(36.3%)		115(14.9%)		61(15.7%)			819(51.9%)	
Partner violence (yes)	101(3.7%)		30(3.9%)		18(4.6%)	0.661		53(3.4%)	0.591
COVID-19 infection in lockdown (yes)	128(4.7%)		27(3.5%)		14(3.6%)	1.000		87(5.5%)	0.043
Chronic diseases (yes)	324(11.8%)		155(20.1%)		54(13.9%)	0.012		115(7.3%)	< 0.0001
Self-rated health (relatively bad)	223(8.1%)		55(7.1%)		24(6.2%)	0.623		144(9.1%)	0.121
Past diagnosis of mental illness (yes)	119(4.3%)		40(5.2%)		24(6.2%)	0.579		55(3.5%)	0.064
Hazardous drinking (yes)	803(29.3%)		195(25.3%)		140(36.0%)	< 0.0001		468(29.7%)	0.031
Smoking (yes)	878(32.1%)		224(29.1%)		90(23.1%)	0.038		564(35.7%)	0.0010
Social capital						0.0020			< 0.0001
P_75_-P_100_	528(19.3%)		181(23.5%)		118(30.3%)			229(14.5%)	
P_50_-P_75_	716(26.2%)		196(25.4%)		117(30.1%)			403(25.5%)	
P_25_-P_50_	723(26.4%)		208(27.0%)		90(23.1%)			425(26.9%)	
≤P_25_	771(28.2%)		186(24.1%)		64(16.5%)			521(33.0%)	

Note: *p* values (non-migrants as reference) were calculated by *χ*^2^ tests for categorical variables and Dunnett-*t* tests for continuous variables

**Fig. 1:**
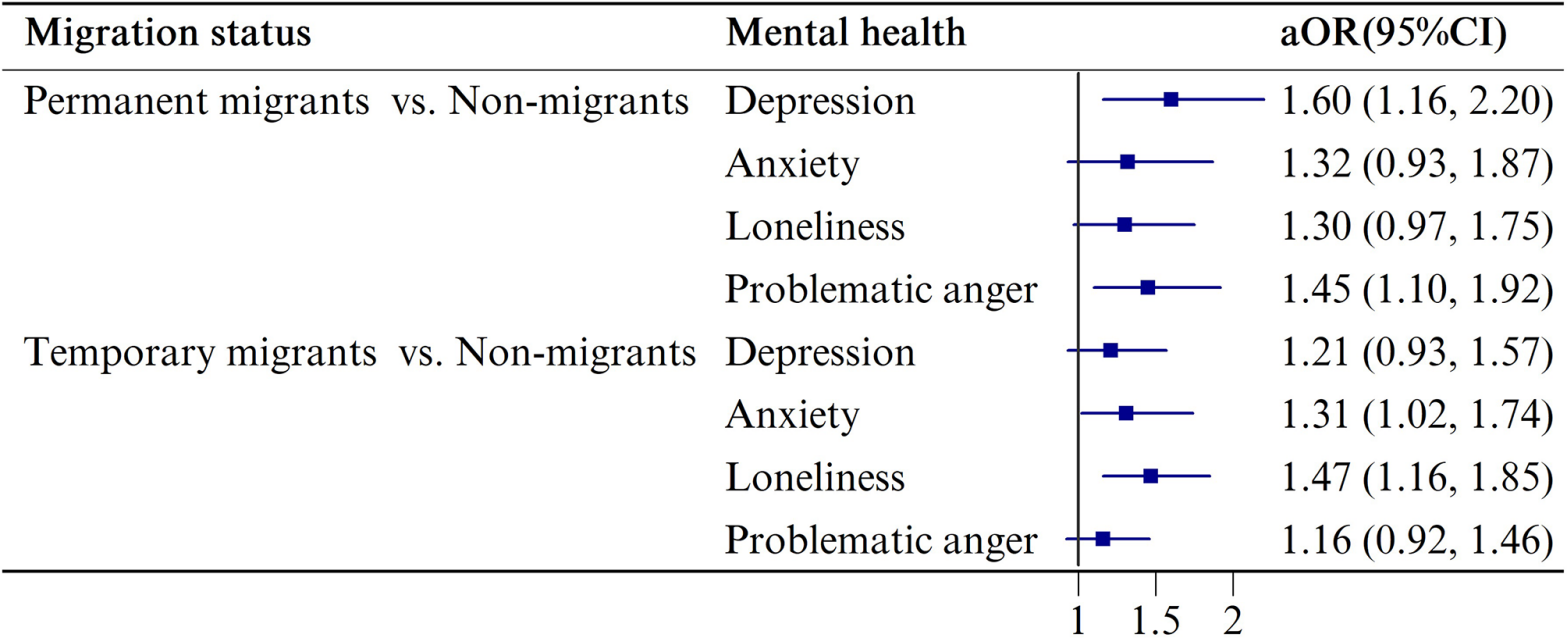
The adjusted associations between migration status and mental health Note: The non-migrants were set as reference group. The associations were adjusted by sets of covariates, which including individual demographics (age, gender, education level, marital status, and employment status), household factors (living arrangement, monthly household income, loss of monthly household income, and partner violence), health-related factors (COVID-19 infection in lockdown, chronic disease, self-rated health, past diagnosis of mental illness, hazardous drinking, and smoking during the lockdown), and social capitals. *aOR*, adjusted odds ratio

The total prevalence of depression, anxiety, problematic anger, and loneliness was 27.3%, 21.0%, 37.4%, and 33.0%, respectively, but participants with different migration statuses showed different prevalences of mental health problems. In general, mental health problems were more prevalent among both permanent and temporary migrants than non-migrants. In comparison with non-migrants (Table 1, depression: 20.6%, anxiety: 16.2%, problematic anger: 32.8%, loneliness: 26.1%), temporary migrants had statistically significantly higher prevalences of depression (30.5%), anxiety (23.5%), problematic anger (38.8%), and loneliness (37.4%), while permanent migrants only had statistically significantly higher prevalences of depression (27.8%) and problematic anger (40.6%). Statistically significant differences in loneliness were only found in temporary migrants (37.4%) when compared to permanent migrants (28.8%). Similar associations were observed in bivariate logistic regression analysis. After adjusting for covariates, permanent migrants continued to have a higher prevalence of depression (adjusted *OR* 1.60 [95% confidence interval (CI) 1.16–2.20]) and problematic anger (1.45 [95% CI 1.10–1.92]) than non-migrants. Temporary migrants had an increased odds of anxiety (1.31 [95% CI 1.02–1.74]) and loneliness (1.47 [95% CI 1.16–1.85]) in adjusted models (Fig. 1).

**Fig. 2:**
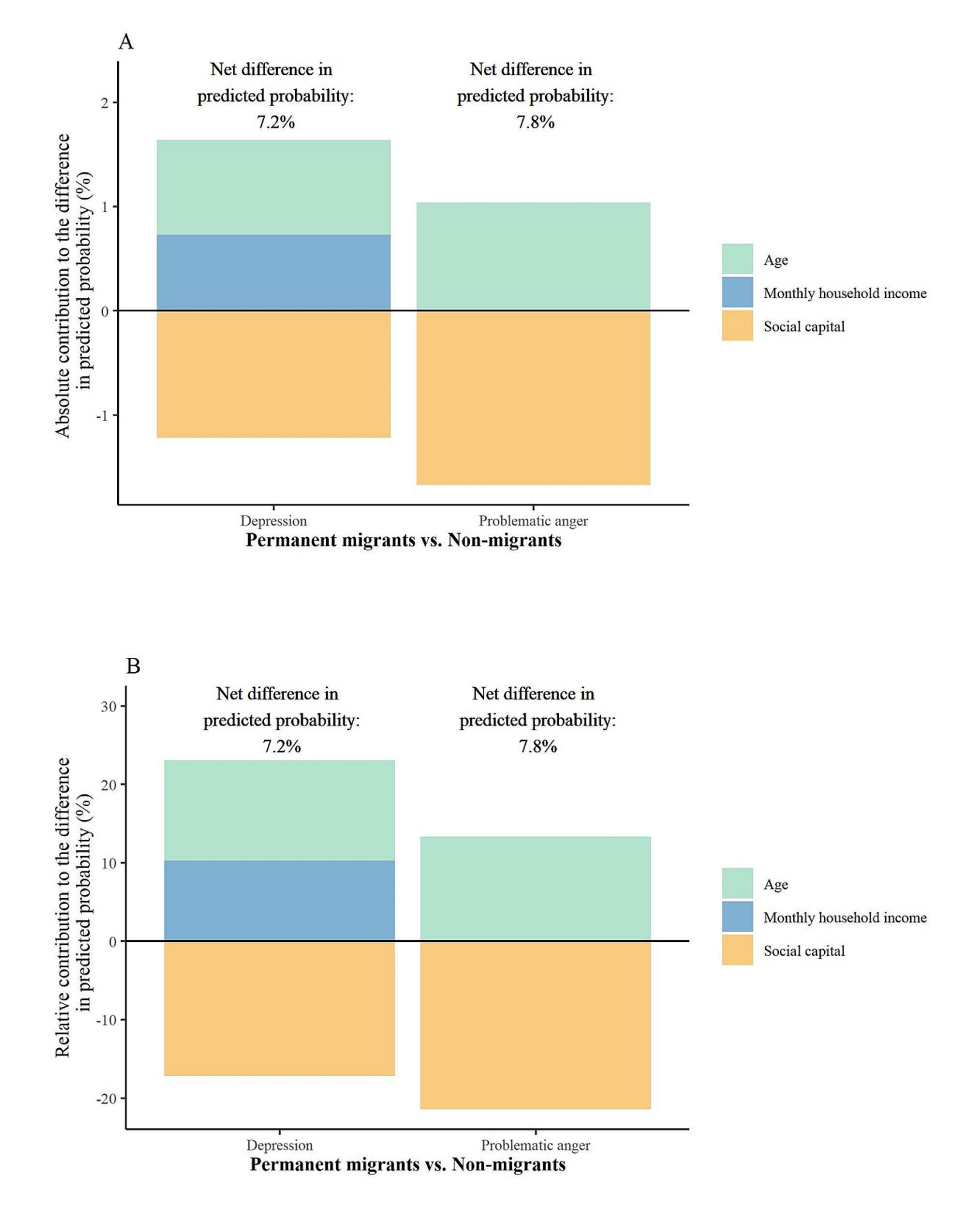
Significant contributors to mental health inequality between permanent migrants and non-migrants A: Absolute contribution for each contributor; B: Relative contribution (proportion of explanation, which equals the value of the absolute contribution divided by net difference) for each contributor. Permanent migrants had a 7.2% (27.8% minus 20.6%, *p* = 0.008) and 7.8% (40.6% minus 32.8%, *p* = 0.019) higher predicted probability of depression and problem anger than non-migrants

**Fig. 3:**
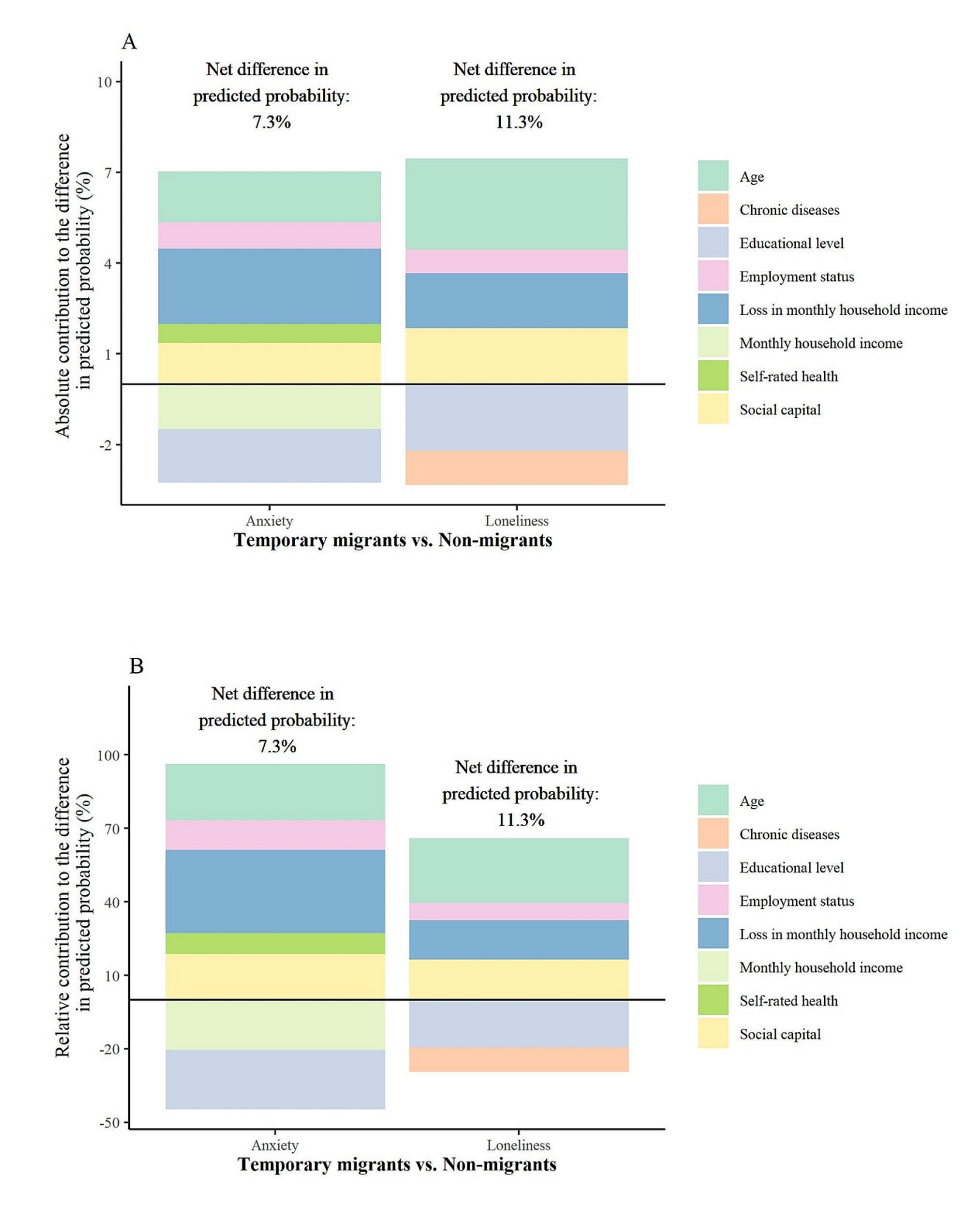
Significant contributors to mental health inequality between temporary migrants and non-migrants A: Absolute contribution for each contributor; B: Relative contribution (proportion of explanation, which equals the value of the absolute contribution divided by net difference) for each contributor. Temporary migrants had 7.3% (23.5% minus 16.2%, *p* = 0.049) and 11.3% (37.4% minus 26.1%, *p* = 0.013) higher predicted probability in anxiety and loneliness than non-migrants

Inequalities in mental health among migrants and non-migrants were further quantified by nonlinear BO decompositions (Figs. 2 and 3). Specifically, significant differences of 7.2% (27.8% minus 20.6%, *p* = 0.008) and 7.8% (40.6% minus 32.8%, *p* = 0.019) in predicted probabilities of depression and problematic anger were observed between permanent migrants and non-migrants (reference group). Significant anxiety and loneliness inequalities were observed between temporary migrants and non-migrants as 7.3% (23.5% minus 16.2%, *p* = 0.049) and 11.3% (37.4% minus 26.1%, *p* = 0.013) respectively.

The quantified contribution of the social determinants to mental health inequalities between permanent migrants and non-migrants is shown in Fig. 2. In Fig. 2A, the magnitude of difference corresponding to each determinant indicates the absolute difference in predicted probabilities statistically explained by each factor (absolute contribution), while the proportion of explanation (relative contribution) was shown in Fig. 2B. For depression, the largest magnitude of the inequality was explained by age, followed by monthly household income, and social capital. Specifically, if permanent migrants had the same distribution in age and household income as non-migrants, the predicted probability of depression would decrease by 0.9% (SD = 0.3%; proportion of explanation: 12.7%) (*p* < 0.05), 0.7% (SD = 0.4%; 10.2%) (*p* < 0.05), respectively. On the contrary, the probability of depression would increase by 1.2% (SD = 0.3%; -17.1%) (*p* < 0.05) if permanent migrants reduced their social capital to the same level as non-migrants.

For problematic anger, similar patterns of contribution were observed for age and social capital. If permanent migrants had the same distribution in age, the probability of problematic anger would decrease by 1.0% (SD = 0.3%; proportion of explanation: 13.3%) (*p* < 0.05), and would increase by 1.7% (SD = 0.4%; -21.4%) (*p* < 0.05) if social capital is the same as non-migrants.

Significant determinants of mental health inequalities between temporary migrants and non-migrants are shown in Fig. 3. For anxiety, age, education level, employment status, loss of income, household income, self-rated health, and social capital significantly contributed to the inequality (*p* < 0.05). The predicted probability of anxiety would be lowered by 2.5% (SD = 0.7%; proportion of explanation: 34.0%), 1.7% (SD = 0.5%; 23.0%), and 1.4% (SD = 0.3%; 18.2%) if temporary migrants had the same distribution of loss of household income, age, and social capital as non-migrants. In contrast, the probability would increase by 1.8% (SD = 0.6%; -24.2%) and 1.5% (SD = 0.5%; -20.4%) if the two groups had the same distribution in education levels and monthly household income.

For loneliness, the predicted probability would lower by 3.0% (SD = 0.7%; 26.5%), 1.9% (SD = 0.3%; 16.3%), and 1.8% (SD = 0.8%; 16.1%) if temporary migrants had the same distribution of age, social capital, and loss of household income as non-migrants, and it would increase by 2.2% (SD = 0.7%; -19.4%) and 1.1% (0.4%, -10.0%) for education level and chronic diseases (*p* < 0.05).

## Discussion

The COVID-19 pandemic, a once-in-a-century public health emergency, exposed and amplified existing health inequalities. Unlike relative normalcy in 2021, the 2022 Shanghai lockdown was abrupt and lasted for months, and left residents inadequately prepared and deficient in essential supplies [31]. This was especially true among marginalized populations like migrants. This study demonstrated mental health inequality among Chinese citizens aged 18 to 59 (except for students) with varied migration statuses and estimated the effects of key social determinants that account for these inequalities. Mental health inequalities were found between non-migrants and internal migrants, but prominent mental health problems differed between permanent and temporary migrants. Several social determinants, including age, household income and income loss, education level, and employment status, and social capital were key contributors to mental health inequalities.

We observed higher estimated prevelances of mental health problems among permanent and temporary migrants compared to non-migrants. There were also differences in the source of mental health burdens among permanent and temporary migrants, where anxiety and loneliness showed higher prevalence in temporary migrants while depression and problematic anger were more prominent among permanent migrants when compared to non-migrants. These differences might result from the varied distribution of common causes of these mental health problems among permanent and temporary migrants. In this study, temporary migrants faced the greatest uncertainty during the lockdown. More than half (51.9%) of them experienced a loss in half their household income, and 19.1% became unemployed due to the lockdown. The major life changes and uncertainty are common causes of both loneliness and anxiety [32]. Meanwhile, temporary migrants also suffered more from financial concerns [33], stressful events, and employment difficulties [34], which are common correlates of anxiety. Overall, this study contributes to the literature on mental health inequality by demonstrating the importance of dividing migrants into groups that reflect their underlying vulnerabilities to mental health problems.

Moreover, we found several key social determinants contributed to mental health inequalities. Age and social capital were significant contributors, but the direction for the two indicators were opposite across the four types of mental health outcomes between non-migrants and migrants. Younger migrants had greater mental health problems in the current study and this aligns with prior studies during COVID-19 [35, 36]. This phenomenon might be explained by more social media use among the youth [37]. We found that younger participants faced more negative social media exposure, such as rumors or insults against Shanghai residents, in our survey (appendix Figure S1). In future public health emergencies, attention should be given to the younger populations, with increased dissemination of positive social media information to this group.

Social capital is an important determinant of inequalities in mental health [5, 38–40]. Consistent with previous studies on Chinese internal migrants [38], temporary migrants reported poorer social capital than non-migrants. Studies on international migrants also support that migration is related to poor social capital [39]. Poor social capital may be associated with marginalization due to registration status (*hukou* status for Chinese internal migrants) and difficulties for migrants in developing social networks and mutual trust in their migration destination [41, 42]. The difference in social capital was positively associated with both the inequality in anxiety and loneliness between temporary migrants and non-migrants. Contrary to previous studies [5, 40], better social capital was observed in permanent migrants compared to non-migrants. Generally, migrants with higher social capital were more likely to have local *hukou* and be permanent migrants [9]. Additionally, we found that the difference in social capital was negatively associated with both the inequality in depression and problematic anger between permanent migrants and non-migrants. In other words, lower reported social capital was associated with greater mental health inequalities. Therefore, a possible interventional pathway to reduce mental health inequality is to enhance the social network and social capital of migrant groups and provide bridges within and between migrants and local residents.

Contrary to expectations, household income was positively associated with inequality in depression between permanent migrants and non-migrants, suggesting higher income was related to higher risks for mental health problems. It was reported that household income is associated with mental health in an inverse-U shape, where extremely high income is positively related with risk of depression [43]. Another possible reason is that migrants with higher income have higher expectations regarding their quality of life, which is harder to achieve because of the lockdown [44]. This suggests that high-income groups also require attention for psychological counseling support. In addition, previous work showed that the contribution of income to health outcomes varies among different socioeconomic groups, suggesting that income might not be a reliable indicator [45]. Notably, temporary migrants endured the most loss of income during the lockdown, and compared to income, loss of household income contributed more to mental health inequalities. This finding supports previous research [46] showing that income loss is a more effective determinant of mental health than increased income, especially for those at a lower level of income. Thus, it is important to ensure financial security of temporary migrants during public health emergencies, governments and social organizations can provide assistance such as unemployment benefits to help temporary migrants cope with crises.

Among the strengths of this study was our nuanced approach to classifying migrants, separating permanent migrants from “non-migrants” by combining *hukou* status and migration experience, which reduced misclassification bias. Another strength is that we quantitatively assessed the social determinants of mental health inequalities. We identified key social determinants that if modified, may reduce mental health inequalities during public health emergencies, providing evidence to support targeted migration-policy.

However, there were some limitations for this study. Limited by the lockdown requirements, an online cross-sectional survey with purposive sampling was practical, though several limitations might be introduced by the study design. First, the cross-sectional design limited causal inference on the impact of the lockdown, but it is worth noting that the social determinants of mental health explored in this research, such as social capital, tended to exhibit relatively stable patterns before and after the lockdown. Moreover, the study incorporated variables related to changes induced by the lockdown, such as unemployment and income reduction. Second, this study was based on the online survey with a non-probabilistic sampling, which might influence representativeness and generalizability. We only included participants aged below 60 to increase the representativeness of migrants and people who exposed to social media. The age and sex distribution of the analytic sample aligns with census [47] and nationwide surveys [48] of internal Chinese migrants. We found a lower total prevalence of mental health problems than was observed in previous national studies [49, 50]. This could be because these studies were conducted during the early stage of the pandemic, when the virus was more toxic and the public knew little about it. Third, there will be inevitable reporting bias as self-report questionnaires were used although we used standardized and validated scales with excellent psychometric properties, and the psychometric properties of self-assessment instruments used in this study have been validated in Chinese population [51, 52]. Additionally, community-level determinants, such as supplies provided by communities in the lockdown, might impact mental health but were not included in this study.

## Conclusion

Significant but heterogeneous inequalities in mental health were found between Shanghai residents with varied migration statuses during an unprecedented COVID-19 lockdown. Compared to non-migrants, mood disorders including depression and problematic anger were higher among permanent migrants while anxiety and loneliness among temporary migrants were of greater concern. Better social capital and economic security contributed significantly to reduce these inequalities. Vulnerability among migrants was seen during the pandemic, and interventions to improve the social determinants of health among the migrant community should be a priority for future policies, and could contribute to achieving Sustainable Development Goal 3 and 10 (improve health and wellbeing & reduce inequality). Population-based interventions aimed at strengthening social capital and economic security (such as unemployment benefits) would likely promote mental health equity between internal migrants and non-migrants during times of crisis, such as this public health emergency.

### Electronic supplementary material

Below is the link to the electronic supplementary material.

Supplementary Material 1

## Data Availability

No datasets were generated or analysed during the current study.
