# Supplementary material for: The determinants of mental health inequalities between Chinese migrants and non-migrants during the Shanghai 2022 lockdown: a Blinder-Oaxaca decomposition
Source: Int J Equity Health. 2024 Jul 9;23:136. doi: 10.1186/s12939-024-02223-2 (PMC11232248; doi:10.1186/s12939-024-02223-2)
Supplement: Supplementary file 1 — Supplementary Material 1 [file 12939_2024_2223_MOESM1_ESM.docx]

**Appendix**

**Title: The determinants of mental health inequalities between Chinese migrants and non-migrants during the Shanghai 2022 lockdown: a Blinder-Oaxaca decomposition**

**Contents**

**[Table S1 Subscales from the Chinese version of Revised Personal Social Capital Scale.](#_Toc147859485)** [2](#_Toc147859485)

[**Supplemental Methods** 3](#_Toc147859486)

**[Figure S1 Distribution of age against exposure to negative information via social media.](#_Toc147859487)** [5](#_Toc147859487)

[**References** 6](#_Toc147859488)

**Table S1 Subscales from the Chinese version of Revised Personal Social Capital Scale.**

| **Survey Question** | | **Scoring** |
| --- | --- | --- |
| **Bonding Social Capital - People in neighborhood** | |  |
|  | How do you rate the number of neighbors you know? | 1=a few, 5= a lot |
|  | Among the neighbors you are familiar with, how many can you trust? | 1=none, 5=all |
|  | Among the neighbors you are familiar with, how many will definitely help you upon your request? | 1=none, 5=all |
|  | Among the neighbors you know, how many of them possess adequate resources, such as very rich, having higher education or a professional job, work for the government, or a CEO in a company? | 1=none, 5=all |
| **Bridging Social Capital - Governmental, political, and social groups and organizations** | |  |
|  | How do you rate the number of these groups and organizations in your community？ | 1=a few, 5= a lot |
|  | Among all these groups and organizations, how many of them represent your rights and interests? | 1=none, 5=all |
|  | Among all these groups and organizations, how many will definitely help you upon request? | 1=none, 5=all |
|  | Among all these groups and organizations, how many have very good reputation in the society? | 1=none, 5=all |

**Supplemental Methods**

The Blinder-Oaxaca (BO) decomposition is a statistical technique to study sources of two-group differences in continuous outcome (linear BO decomposition). ^1^ It was originally proposed to measure the difference in wage between men and women, and quantify the significant contribution of a set of variables to the inequality based on regression models in a counterfactual manner. ^2,3^ As an extension of the method to category outcome (non-linear BO decomposition), the method has also been widely used in epidemiology and health-related studies. ^4-6^ We adopted the two-fold non-linear BO decomposition widely used in discrimination literature because of binary outcomes in this study.^7^

There are two steps to decompose the group-differences, which are described mathematically by equations (1) - (4). Firstly, we calculated the predicted probability of mental health problems by estimating a logistic regression model for each group (Equation 1-2),

1. $\bar{Y_{A}}=F\left( X_{A}\hat{\beta}_{A} \right)$
2. $\bar{Y_{B}}=F\left( X_{B}\hat{\beta}_{B} \right)$

Where A refers to permanent/temporary migrant group, B refers to non-migrant group (reference group). *F* is the cumulative distribution function of logistic regression. $\bar{Y}_{A}/\bar{Y}_{B}$ is the estimated predicted probability.

Secondly, mental health inequality is quantified and decomposed into two components by equation 3, which is calculated based on logistic regressions described in equation 1-2 in a counterfactual manner. $\bar{Y}_{A}-\bar{Y}_{B}$ is the difference in average probability of mental health problem between two groups, i.e., permanent/temporary migrants versus non-migrants, quantifying the mental health inequality. The group difference is decomposed into two components: the explained component (denoted by *E* in equation 4) which captures inequality that can be explained by the differences in the levels of observed characteristics (including individual demographics, household factors, health-related factors, and social capital), and the unexplained component (denoted by *U* in equation 5) which represents the inequality explained by the differential effect of observed factors and both differential levels and effects of unobserved factors.

1. $\bar{Y}_{A}-\bar{Y}_{B}=\left[ \sum_{j=1}^{N_{A}} \frac{F\left( X_{Aj}\hat{\beta}^{*} \right)}{N_{A}}-\sum_{j=1}^{N_{B}} \frac{F\left( X_{Bj}\hat{\beta}^{*} \right)}{N_{B}} \right]+\left[ \sum_{j=1}^{N_{B}} \frac{F\left( X_{Bj}\hat{\beta}_{A} \right)}{N_{B}}-\sum_{j=1}^{N_{B}} \frac{F\left( X_{Bj}\hat{\beta}_{B} \right)}{N_{B}} \right]$, where
2. $E=\sum_{j=1}^{N_{A}} \frac{F\left( X_{Aj}\hat{\beta}^{*} \right)}{N_{A}}-\sum_{j=1}^{N_{B}} \frac{F\left( X_{Bj}\hat{\beta}^{*} \right)}{N_{B}}$ , and
3. $U=\sum_{j=1}^{N_{B}} \frac{F\left( X_{Bj}\hat{\beta}_{A} \right)}{N_{B}}-\sum_{j=1}^{N_{B}} \frac{F\left( X_{Bj}\hat{\beta}_{B} \right)}{N_{B}}$

In a counterfactual manner, change in the predicted probability (absolute contribution) is calculated by replacing one social determinant of the reference group (non-migrants) with that of a migrant group (permanent or temporary migrants), while fixing the distribution of other determinants in the model (denoted by *U* in equation 5). The proportion of explanation (relative contribution) is calculated by dividing the change in predicted probability by the group-difference in predicted probabilities. The sign of the proportion of explanation indicates the direction of the contribution, where a negative sign indicates a negative contribution, meaning that removing the intergroup differences in this factor will increase the inequality, and vice versa.

$\hat{\beta}^{*}$is reference coefficient which defined a nondiscriminatory condition. It is valued between $\hat{\beta}_{A}$ and $\hat{\beta}_{B}$, but it is not clear which regression coefficients should be selected as reference. In this study, we adopted the pooled coefficient as reference according to Neumark’s suggestion.^8^

The decomposition analysis was performed using the Stata “fairlie” command, which provides non-linear predictions.^9^ Pooled coefficient was set as reference by option “pooled”. Meanwhile, the method requires a one-to-one matching of the observations from two group. In this study, there are different sample sizes in each group. To address the problem, we draw a random subsample of group with greater sample size for 1000 replication using the option “reps” in the “fairlie” package. Non-linear decomposition results based on logistic regressions may vary depending on the order of independent variables being included, so we randomly ordered the variables across replications of subsampling. The results of decomposition were displayed in figure 1-2.


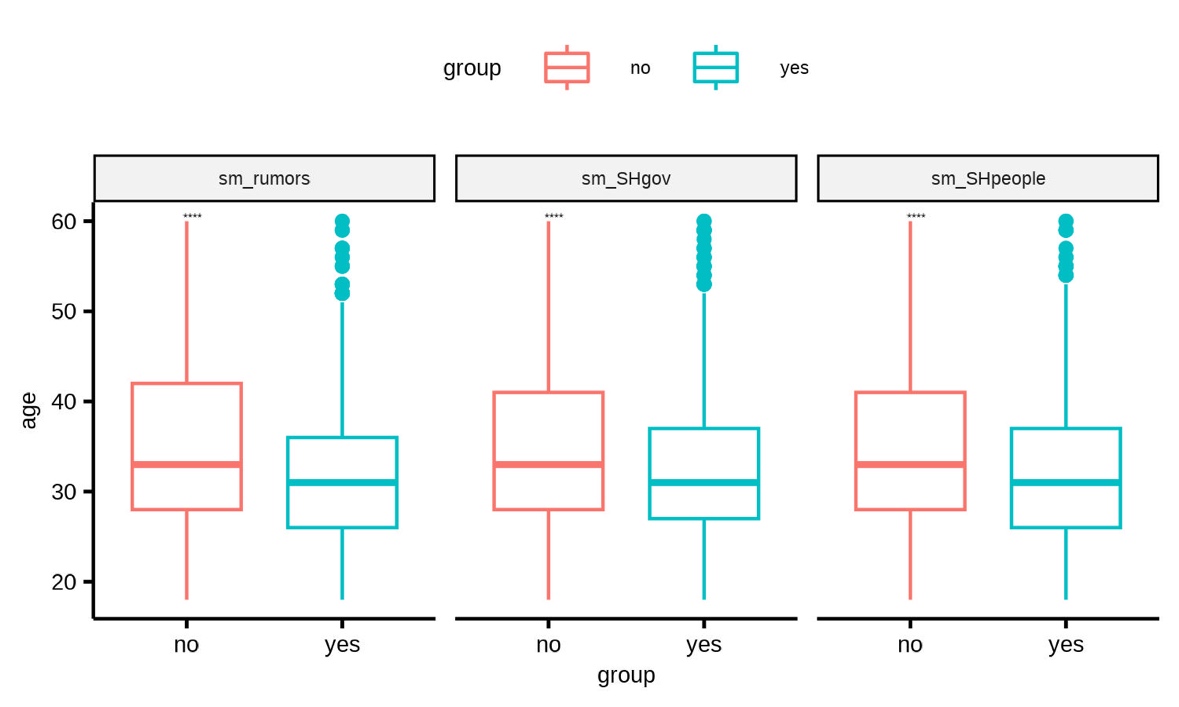
 **Figure S1 Distribution of age against exposure to negative information via social media.**

sm_rumors: Have you seen any rumors about lockdown and quarantine in Shanghai on social media? sm_SHgov: Have you been exposed to unfriendly posts/comments towards Shanghai government on social media?

sm_SHpeople: Have you been exposed to unfriendly posts/comments towards Shanghai citizens on social media?

****: *p*-value <0·0001 with *t*-test.

**References**

1. Jann B. The Blinder–Oaxaca Decomposition for Linear Regression Models. *The Stata Journal* 2008; **8**(4): 453-79.

2. Blinder AS. Wage Discrimination: Reduced Form and Structural Estimates. *The Journal of Human Resources* 1973; **8**(4): 436-55.

3. Oaxaca RL. Male-Female Wage Differentials in Urban Labor Markets. *International Economic Review* 1973; **14**: 693-709.

4. Mehta HB, Rajan SS, Aparasu RR, Johnson ML. Application of the nonlinear Blinder-Oaxaca decomposition to study racial/ethnic disparities in antiobesity medication use in the United States. *Research in social & administrative pharmacy : RSAP* 2013; **9**(1): 13-26.

5. Shah NS, Huang X, Petito LC, et al. Social and Psychosocial Determinants of Racial and Ethnic Differences in Cardiovascular Health in the United States Population. *Circulation* 2023; **147**(3): 190-200.

6. Allen J, Cotter-Roberts A, Darlington O, Dyakova M, Masters R, Munford L. Understanding health inequalities in Wales using the Blinder-Oaxaca decomposition method. *Front Public Health* 2022; **10**: 1056885.

7. Oaxaca RL, Ransom MR. Identification in Detailed Wage Decompositions. *The Review of Economics and Statistics* 1999; **81**(1): 154-7.

8. Neumark D. Employers' Discriminatory Behavior and the Estimation of Wage Discrimination. *Journal of Human Resources* 1987; **23**: 279-95.

9. Jann B. FAIRLIE: Stata module to generate nonlinear decomposition of binary outcome differentials. *Statistical Software Components* 2006.
